# Supplementary material for: Screen time, sleep quality and mental health among adolescents of secondary schools in Dharan
Source: PLOS Ment Health. 2025 Nov 7;2(11):e0000405. doi: 10.1371/journal.pmen.0000405 (PMC12798179; doi:10.1371/journal.pmen.0000405)
Supplement: Attachment — Submitted filename: Response to Reviewers.docx [file pmen.0000405.s002.docx]

22^nd^ September 2025

To,
PLOS Mental Health,

Dear Editor,

We sincerely thank you and the reviewers for the constructive feedback on our manuscript entitled “Screen time, sleep quality and mental health among adolescents of secondary schools in Dharan” (PMEN-D-25-00306). We have carefully considered all comments and made substantial revisions to improve clarity, completeness, and scientific rigor. Below we provide a consolidated response to the issues raised.

**Data Availability**

We acknowledge the journal’s policy on data sharing. We would like to clarify that all data underlying the findings in our study have already been fully included in the manuscript itself in the form of tables. The dataset used for analysis contained only the same data points presented in the manuscript, and no additional raw dataset exists beyond what has been reported. Since all values used for calculations (including descriptive statistics and frequencies) are fully presented in the manuscript, other researchers can reproduce and verify our results directly from the published data. Therefore, we believe our study fulfills the PLOS requirement of data being made available within the manuscript itself. This ensures transparency and reproducibility while respecting ethical requirements and protecting participant confidentiality.

**Abstract and Grammar**

The abstract has been revised for clarity and grammatical correctness. P-values have been added to highlight key findings, and typographical errors (e.g., “scree time” → “screen time,” “screen quality” → “sleep quality”) have been corrected.

**Introduction**

The introduction has also been extended to give definitions of mental health and sleep and how they relate to each other among the adolescents. Operational definitions of screen time, sleep quality and mental health are also included. The weight of the issue has been put forward in a more understandable manner, with global, regional, and local evidence involved. The already existing literature about screen time and adolescent mental health is being mentioned now, and the gap in the knowledge that our study fills is clearly defined. Some brief description of physiological processes that are associated with exposure to screens disrupting sleep and mental health (e.g. melatonin suppression) has also been provided.

**Method**

The method section has been revised extensively for transparency. We have deleted the redundant use of “design.” Eligibility criteria are now clearly stated, and the rationale for including adolescents aged 12–19 years is explained. The sampling technique has been revised to clarify the multi-stage procedure, specifying how students were systematically selected from the identified schools, including the use of systematic random sampling, calculation of the k-value, selection of the random starting point, and ensuring proportional representation across all school grades. The sample size calculation has been fully explained, including prevalence assumptions and non-response adjustment.

Descriptions and references for all instruments (STQ, PSQI, PHQ-9) have been added with proper references. The cut-offs used to define “good” and “poor” sleep quality (PSQI score 0–5 vs. ≥6) and “no/mild” vs. “depression” (PHQ-9 score 0–9 vs. ≥10) are now explicitly reported. The categorization of explanatory variables (e.g., screen time ≤2 vs. >2 hours/day; socio-demographic variables) has also been detailed. Justification for the use of SPSS v11.5 is provided, noting that it was the licensed version available institutionally at the time.

**Results**

The presentation of results has been refined for clarity and consistency. Age range starting from 12 years has been justified. Ethnic categories in Table 1 are standardized as of Nepal Census Survey. Also, the cut-offs for “high” and “low” screen exposure have been mentioned, and odds ratios in respective table are clarified as unadjusted. Redundancy between text and tables has been reduced.

**Discussion**

Differences between the students of the public and the private schools have been explained, which has added more weight to the discussion, taking into consideration the socio-environmental and academic factors. We have increased comparisons with international. The negative effects of spending too much time on a screen is explained on the background of the latest research. To enhance the discussion of digital and interactive methods of adolescent health literacy, the proposed source (Mancone et al., 2024) has been incorporated.

**Limitation**

An appropriate Limitations section has now been added to the manuscript, covering the cross-sectional design, the use of self-reported measures, and the lack of generalizability of results because of the study being conducted in one city.

**Conclusion**

The conclusion has been revised for clarity, correcting errors and adding practical implications for schools, parents, and policymakers, including recommendations for awareness campaigns, prevention strategies, and targeted interventions.

We are grateful for the reviewers’ insightful feedback, which has allowed us to substantially improve our manuscript. We trust that the revisions adequately address all concerns, and we hope the revised version will be considered suitable for publication.

***Sincerely,***

Vivek Gyawali

On behalf of all authors
